# Detwinning through migration of twin boundaries in nanotwinned Cu films under *in situ* ion irradiation

**DOI:** 10.1080/14686996.2018.1428877

**Published:** 2018-03-02

**Authors:** Jinlong Du, Zaoming Wu, Engang Fu, Yanxiang Liang, Xingjun Wang, Peipei Wang, Kaiyuan Yu, Xiangdong Ding, Meimei Li, Marquis Kirk

**Affiliations:** ^a^ State Key Laboratory of Nuclear Physics and Technology, School of Physics, Peking University, Beijing, P.R. China; ^b^ State Key Laboratory of Advanced Optical Communication Systems and Networks, Peking University, Beijing, P.R. China; ^c^ Department of Materials Science and Engineering, China University of Petroleum, Beijing, P.R. China; ^d^ State Key Laboratory for Mechanical Behavior of Materials, Xi’an Jiaotong University, Xi’an, P.R. China; ^e^ Nuclear Engineering Division, Argonne National Laboratory, Argonne, IL, USA

**Keywords:** *In situ*, ion irradiation, nanotwins, detwinning, 10 Engineering and Structural materials, 102 Porous / Nanoporous / Nanostructured materials, 106 Metallic materials

## Abstract

The mechanism of radiation-induced detwinning is different from that of deformation detwinning as the former is dominated by supersaturated radiation-induced defects while the latter is usually triggered by global stress. *In situ* Kr ion irradiation was performed to study the detwinning mechanism of nanotwinned Cu films with various twin thicknesses. Two types of incoherent twin boundaries (ITBs), so-called fixed ITBs and free ITBs, are characterized based on their structural features, and the difference in their migration behavior is investigated. It is observed that detwinning during radiation is attributed to the frequent migration of free ITBs, while the migration of fixed ITBs is absent. Statistics shows that the migration distance of free ITBs is thickness and dose dependent. Potential migration mechanisms are discussed.

## Graphical abstract

**Figure:**
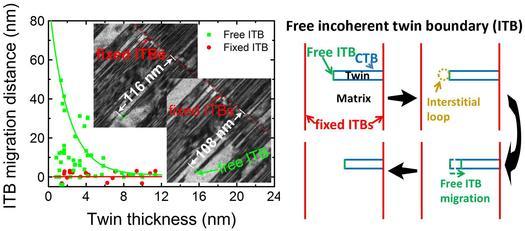

## 1. Introduction

The nanotwinned (NT) metals have attracted much attention as the twin boundaries (TBs) in NT metals underscore their unique combination of properties such as high strength, high ductility, excellent thermal stability, and retained electrical conductivity [1–6]. Besides, NT metals have shown superior microstructural stability under ion irradiation due to the existence of high density coherent TBs (CTBs) [7,8]. It has been proposed that the partial dislocations at the CTBs can unlock the stair-rod dislocations of stacking fault tetrahedra (SFTs), resulting in the removal of SFTs [7,9–11]. Other *in situ* studies have shown that CTBs could accelerate the recombination of unlike defects by rapid transportation of interstitials along TBs to the region with high vacancy concentration, enabling the shrink of preexisting nanovoids [12].

On the other hand, the incoherent twin boundaries (ITBs) in NT metals are found to be unstable and easy to migrate during ion irradiation and deformation, especially when the twin thickness is smaller than 10 nm [5,8,13–15]. Previous studies reported that ITBs can migrate under ultra-low stress in *in situ* nanoindentation studies [15,16], or migrate by absorbing self-interstitial atoms or vacancies in *in situ* heavy ion irradiation studies, which may lead to the detwinning of NT metals [8,13].

The mechanism of deformation-induced detwinning could be understood from two cases. The first case is accomplished via the collective glide of Shockley partial dislocations at ITBs, and the migration of ITBs may lead to the detwinning of NT metals [16–23]. The second case is accomplished via dislocation climb by absorbing interstitials or vacancies, causing the TBs to migrate back and forth [19–21].

Recently, several studies have revealed that detwinning during radiation is due to the migration of ITBs and the migration tendency is related to the stacking faults energy and twin thickness [8,13]. It was reported that the ITBs with twin thickness of a few nanometers are easy to migrate during ion irradiation and the migration rate of ITBs decreases progressively with the increase in twin thickness [8].

In this work, we find two types of ITBs with different migration abilities during heavy ion irradiation. *In situ* results show that a large fraction of ITBs can migrate freely and the migration velocity is sensitive to twin thickness and dose. In contrast, some ITBs are immobile even if the twin thicknesses are only a few nanometers. Discussions on the migration mechanisms might provide further understanding of the detwinning mechanism of NT metals during ion irradiation.

## 2. Experimental details

A Cu (99.99%) target was used to deposit ~2μm-thick Cu films on 10% HF-etched Si (110) substrate by magnetron sputtering approach with a deposition rate of 0.5 nm/s. The chamber was evacuated to a base pressure less than 2 × 10^−5^ Pa prior to deposition. The substrate was heated to and kept at 100 °C during deposition. The cross-sectional TEM (XTEM) specimens were prepared by dimpling and low energy (3.5 keV) Ar ion milling and subsequent ion polishing [24,25]. Before and after radiation, the specimen was investigated by an FEI Tecnai F20 microscope (FEI, Hillsboro, OR, USA). *In situ* 1 MeV Kr^2+^ ion irradiation was performed at room temperature at intermediate voltage electron microscope (IVEM) Tandem facility at Argonne national laboratory, where a Hitachi H-9000NAR microscope (Hitachi, Tokyo, Japan) is attached to an ion implanter. The dose rate during *in situ* ion irradiation experiment was 1.59 × 10^−4^ dpa/s. The microscope was operated at 200 kV and recorded the microstructural evolution during ion irradiation. The CCD camera captured images at 15 frame s^−1^. The radiation damage profile in unit of displacements per atom (dpa) was calculated using SRIM (Kinchin–Pease method) [26] and the damage values in this article were taken as the average values of the radiation damage in the first 100 nm radiation damage profile (TEM foil thickness is about 100 nm). Most Kr ions (99.99%) penetrated through the thin area (around 100 nm thick) of TEM specimen due to high ion energy of 1 MeV.

## 3. Results and discussion

**Figure 1.**
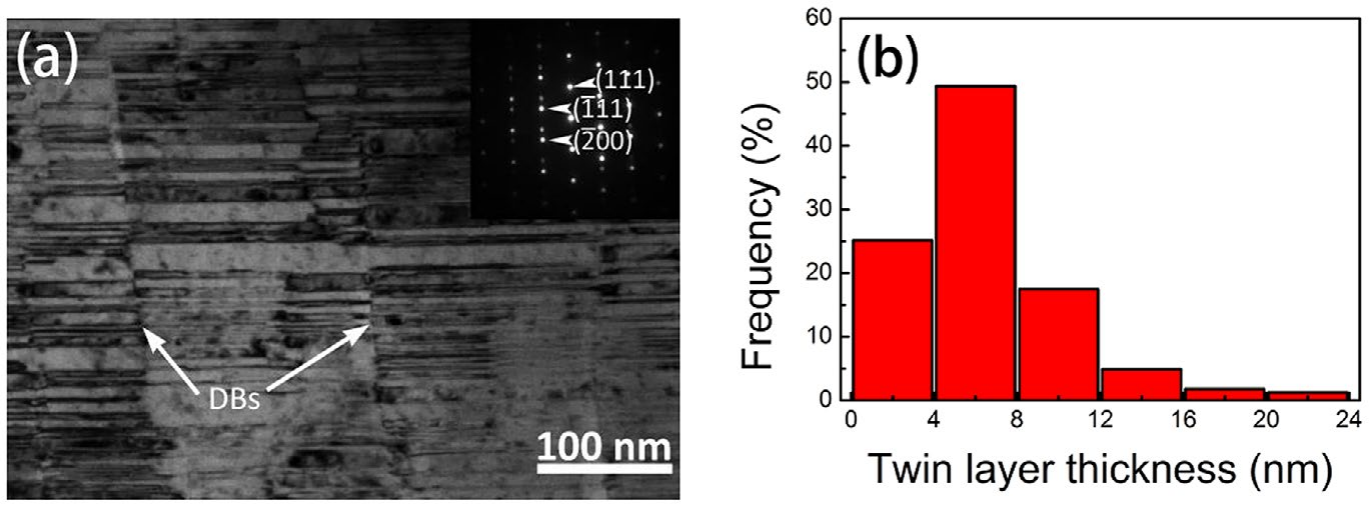
(a) Bright-field (BF) XTEM micrograph with [01$\overset{¯}{1}$] zone axis and (b) the statistic graph of twin thickness of the as-deposited NT Cu film.

Figure 1(a) shows the bright-field (BF) XTEM micrograph of as-deposited NT Cu film taken by tilting the specimen to [01$\overset{¯}{1}$] zone axis. Upper-right inset is the corresponding selected area diffraction (SAD) image, which indicates the single-crystal Cu film along [01$\overset{¯}{1}$] zone axis with spot splitting across the {1 1 1} twin plane. The as-deposited Cu film has a columnar structure and a high density of {1 1 1}-type twin interface oriented parallel to the substrate surface. The statistical average twin thickness (*t*) was about 6 ± 2 nm (as Figure 1(b) shows), which is similar to the matrix thickness. The column width was 100 ± 20 nm, and the domain boundaries (DBs) are labeled by the white arrows. Before radiation, the TEM sample is tilted to [01$\overset{¯}{1}$] zone axis parallel to the direction of electron beam.

**Figure 2.**
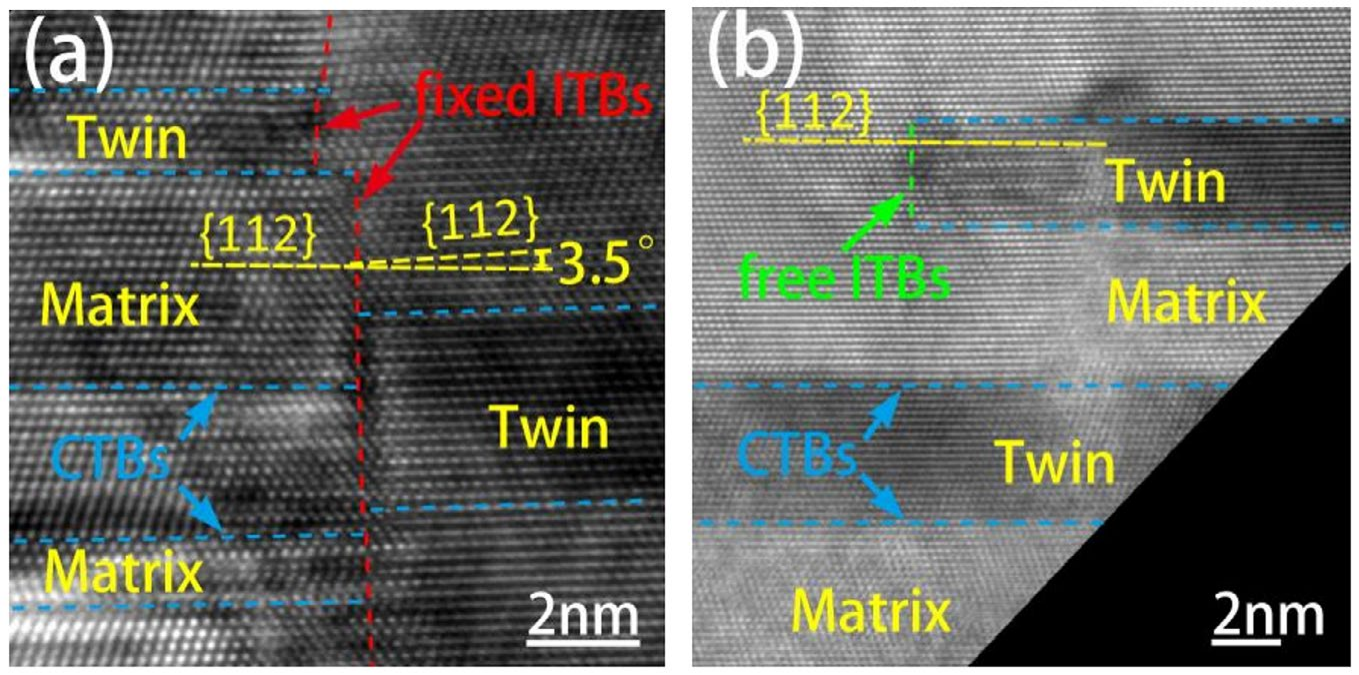
HRTEM micrographs of (a) fixed ITBs labeled by a red dash line and (b) free ITBs labeled by a green dash line in NT Cu film. The lateral {1 1 1} CTBs are labeled by sky-blue dash lines. Lateral {1 1 2} planes across fixed ITBs are misaligned by 3.5°.

Figure 2 shows the high-resolution TEM (HRTEM) micrographs of two types of ITBs defined according to their structural features. The gray and dark areas correspond to the matrix and twin, and the lateral {1 1 1} CTBs are labeled by sky-blue dash lines. Two types of {1 1 2} ITBs are defined and they are labeled by red dash lines in Figure 2(a) and green dash lines in Figure 2(b), respectively. The first type of ITBs (labeled by red dashed lines) is shown in Figure 2(a), and they are continuous between columnar grains or end in the CTBs. We named these ITBs as ‘fixed ITBs’. In fact, the fixed ITBs usually form the DBs between grains in NT Cu film. As they are located in different crystal columns, the lateral {1 1 2} planes across the fixed ITBs are mostly misaligned by a small angle. For example, the misaligned angle between two {1 1 2} planes across fixed ITBs shown in Figure 2(a) is about 3.5° labeled by yellow dash lines in Figure 2(a). Another type of ITB is called free ITBs shown as Figure 2(b). The free ITB separated by a matrix grain and labeled by a green dash line has a length of 2.7 nm in Figure 2(b). Due to the boundary of the twin lamella being surrounded by the matrix, the lateral {1 1 2} planes coincide in the adjacent grains, and the free ITBs are mostly located inside the matrix grains. In some cases, twin lamellae terminating at DBs also exhibit the same structure, and they are free ITBs too. The radiation response of these two types of ITBs with different structures under the same ion irradiation is investigated in the following.

During heavy ion irradiation in face-centered cubic (fcc) metals such as Ag, Au, Cu, and Ni with low-to-intermediate stacking faults energy, a large number of interstitials and vacancies are generated and developed to interstitial loops and SFTs, which are primarily defect clusters in radiated fcc metals [7,13,27–29]. Recent studies showed that the interstitial loops induced by the *in situ* Kr ion irradiation could be easily captured by the ITBs during radiation process at room temperature and the radiation leads to the migration of ITBs [7,8,13,30].

**Figure 3.**
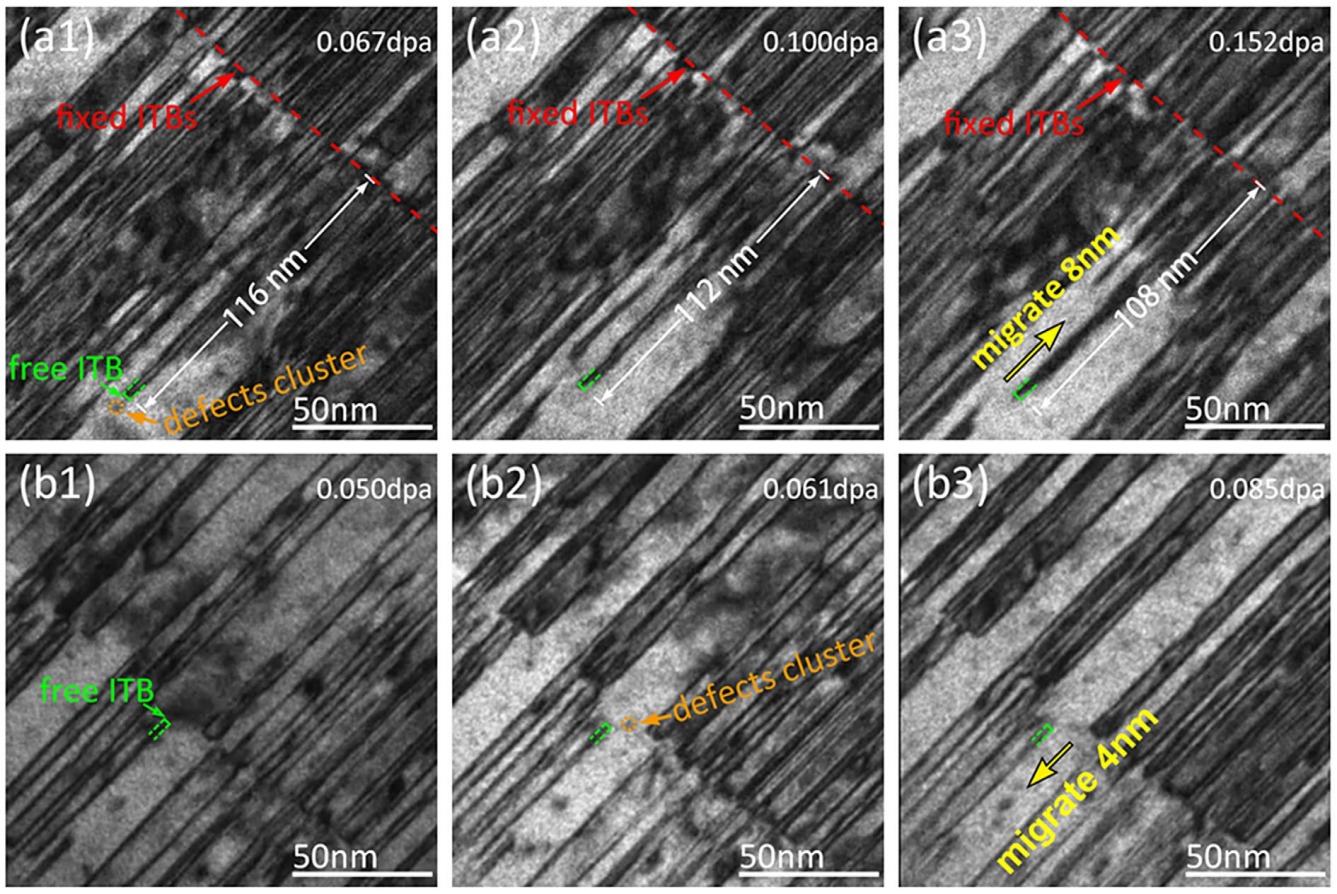
A series of BF TEM snapshots of irradiated Cu film were captured over a dose range of (a1–a3) 0.067–0.152 dpa and (b1–b3) 0.050–0.085 dpa. Fixed ITBs are labeled by a red dash line, and free ITB is labeled by a green dash line. Free ITBs migrating along opposite directions are observed. (a1–a3) The free ITB with the twin thickness of 4 nm migrates 8 nm to upper right direction by absorbing the radiation-induced defects clusters. (b1–b3) The free ITB with twin thickness of 3 nm migrate 4 nm, to lower left direction, by absorbing radiation-induced defects clusters. The figures without any annotations and optional lines are listed as the supplementary material.

Figure 3 shows a series of BF TEM snapshots of *in situ* ion-irradiated NT Cu film over a dose range of (a1–a3) 0.067–0.152 dpa (see Supporting Video V1) and (b1–b3) 0.050–0.085 dpa. The migration of fixed ITBs, which are labeled by red lines in Figure 3(a1–a3), was not observed during ion irradiation, and furthermore, no migration of fixed ITBs was observed in the whole specimen. The free ITB was labeled by a green line in Figure 3. In Figure 3(a1–a3): before radiation, the twin thickness (*t*) is 4 nm, and the length of twin lamella is 116 nm. The twin lamella is surrounded by a matrix with thickness of 30 nm. The average size of the defect clusters (typical one is labeled by orange arrow) was measured to be 7 ± 1 nm. Migration of this free ITB is observed and it is caused by absorbing the radiation-induced defect clusters. The migration distance is about 8 nm according to the reduction of the twin lamella’s length from 116 nm (Figure 3(a1)) to 108 nm (Figure 3(a3)), that is, the free ITB migrates 8 nm, to upper right direction. (b1–b3) shows that a free ITB with twin thickness of 3 nm migrates 4 nm, toward the lower left direction. Comparing the snapshots of (b1–b3) with (a1–a3), a free ITB migrating along an opposite direction is observed. Previous studies showed that the grain boundaries (GBs) could interact with both Frank loops [30] and SFTs [7], and thus a large number of Frank partial dislocations may exist along ITBs. These existing Frank partial dislocations might absorb self-interstitial atoms, causing the ITB to migrate toward the right-upper, or absorb vacancies, causing the ITB to migrate left-downwards. The migration mechanism of the free ITBs will be discussed below.

**Figure 4.**
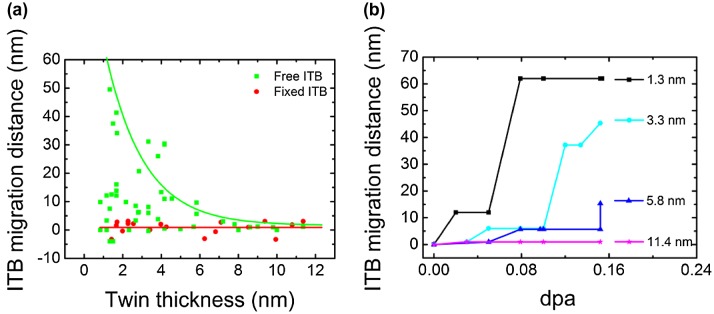
The dependence of the migration distance of free and fixed ITBs on twin thickness. (a) Migration distance of free ITBs decreases with the increase in the twin thickness over the dose range of 0–0.152 dpa. The fixed ITBs (red) are more stable than the free ITBs (green). (b) The plot of the migration distance of four free ITBs over radiation dose range of 0–0.152 dpa for twins with thickness of 1.3–11.4 nm. It shows that the migration distance of free ITBs increases with dose, and also free ITBs with thicker twin thicknesses need higher dpa before migration.

Figure 4(a) shows the migration behaviors of free ITBs and fixed ITBs vs. twin thickness over the radiation dose of 0–0.152 dpa. The twin thickness of as-deposited NT Cu film ranges from 1 to 12 nm. The migration distance of fixed ITBs plotted by the red dots in Figure 4(a) is negligible. Migration distance of free ITBs plotted by the green dots in Figure 4(a) is related to the twin thickness. It is found that free ITBs’ migration distance decreases with the increase in twin thicknesses and they are stable when the twin thickness is larger than 8 nm.

Figure 4(b) shows the plot of migration distance of free ITBs over radiation dose of 0–0.152 dpa for 4 pieces of twin lamellae with twin thicknesses of 1.3 ~11.4 nm. It shows that the migration distance of free ITBs increases with the increase in dpa. The ITB with twin thickness of 1.3 nm first migrates 12 nm when radiation dose reaches 0.02 dpa without incubation, and then it has an incubation period and keeps static before damage increases to 0.06 dpa. After that, the ITB migrates 60 nm from 0.06 to 0.08 dpa, and then remains stable. The ITBs with twin thicknesses of 3.3 and 5.8 nm exhibit similar migration behaviors.

To understand the reason why fixed ITBs and free ITBs exhibit different behaviors under radiation, it is necessary to understand the ITBs’ migration mechanism [13]. Two ITBs’ migration mechanisms: glide of Shockley partial dislocations and climb of Frank partial dislocations, have been reported. First, the ITBs’ migration can be a result of the collective glide of partial dislocations. It is reported that the ITBs typically consist of an array of mobile Shockley partial dislocations [16]. ITBs can migrate through stress-driven glide of mobile Shockley partial dislocations. Second, it is found that the perfect dislocation loops could be absorbed by ITBs, following the reaction of $\frac{1}{2}\left[\overset{¯}{1}\overset{¯}{1}0\right]+\frac{1}{6}\left[112\right]\to \frac{1}{3}\left[\overset{¯}{1}\overset{¯}{1}1\right]$, and lead to the formation of Frank partial dislocations in ITBs [13]. Thus, it is likely that ITBs could migrate via dislocation climb by absorbing self-interstitial atoms or vacancies. In fact, both these migration behaviors have been reported in *in situ* radiation experiments [13,16].

Figure 4(a) shows that the migration distance of free ITB depends on twin thickness, and the smaller the twin thickness, the larger the migration distance. It is also found that some free ITBs with thickness of smaller than 3 nm exhibit no obvious migration behaviors. We hold the view that when the twin thickness is smaller than 3 nm, ITBs migrate mainly through stress-driven glide of mobile Shockley partial dislocations. Stress arising from radiation process leads to the ITBs’ migration; thus, some ITBs migrate when subjected to stress and some ITBs exhibit unobvious migration with no stress applied.

As twin thickness increases, ITBs migrate via both the stress-driven glide of mobile Shockley partial dislocations and dislocation climb by absorbing self-interstitial atoms or vacancies. Previous studies [13] showed that the driving force for ITBs’ migration is the reduction of potential energy via recombination of vacancies and interstitials. Also, we know that the energy of Frank partial dislocations’ climb is much larger than the energy of Shockley partial dislocations’ glide. For free ITBs, as the twin thickness increases, ITBs migrate via both the stress-driven glide of mobile Shockley partial dislocations and dislocation climb by absorbing self-interstitial atoms or vacancies.

Free ITBs first migrate by the climb of Frank partial dislocations when they are absorbing defect clusters and the free ITBs turn to the stepped grain boundary, which includes both the formed free ITBs and CTBs [13]. Then, the formed free ITBs could glide subjected to stress. As the twin thickness increases, more climb behaviors of Frank partial dislocations are needed, thus free ITBs with larger twin thicknesses exhibit smaller migration distances.

**Figure 5.**
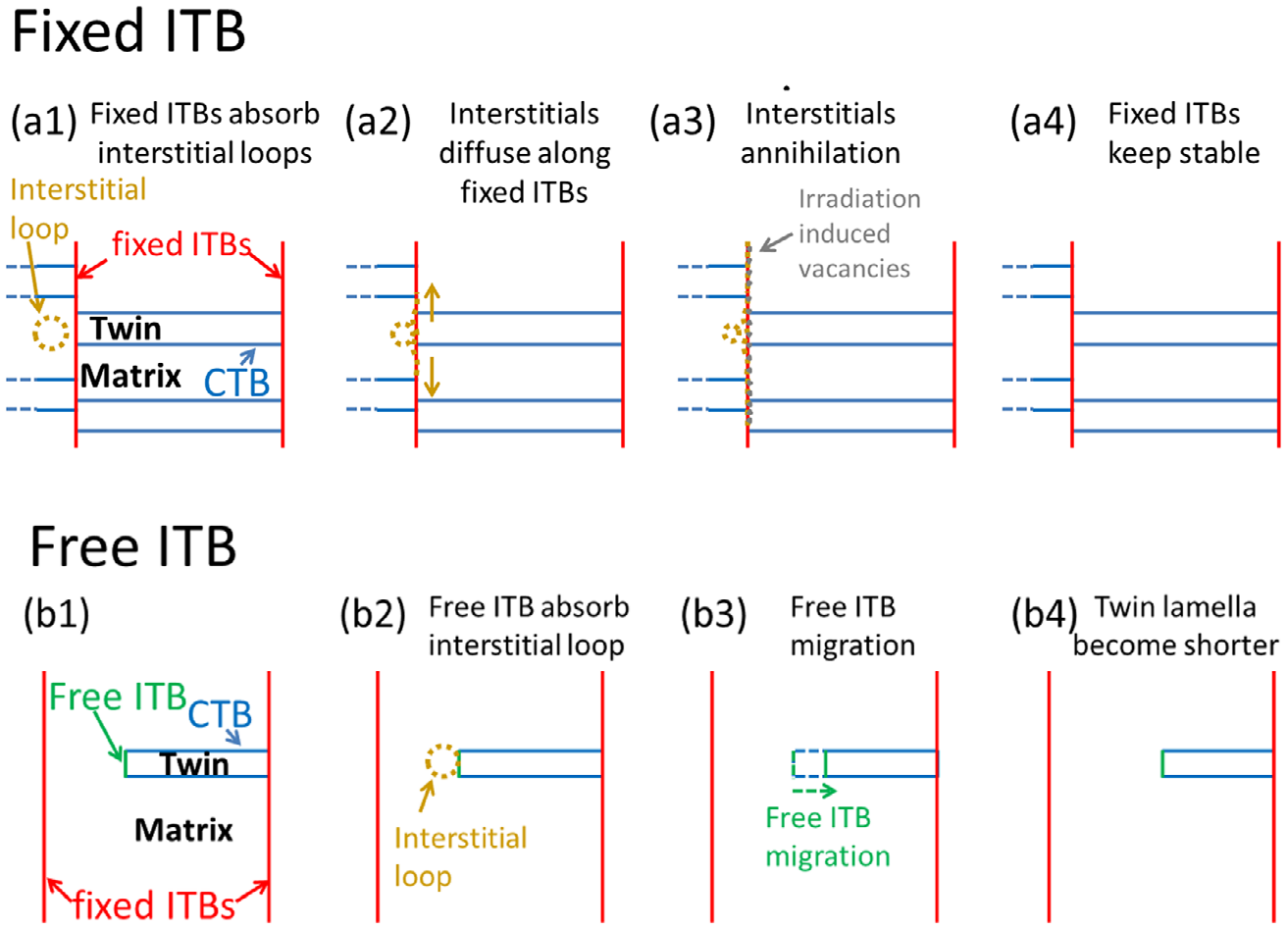
Schematic figures of the interaction between ITBs and radiation-induced interstitial loops. (a1–a4): fixed ITBs interact with radiation-induced defect clusters; (a1) fixed ITBs labeled by red solid lines absorb interstitial loop; (a2) absorbed interstitials diffuse along fixed ITBs; (a3) the high-density interstitials annihilated by absorbing radiation-induced vacancies and vacancy clusters; and (a4) fixed ITB keeps stable. (b1–b4): free ITBs interact with radiation-induced interstitial loops; (b1) the free ITB is labeled by a green line; (b2) the free ITB absorbs radiation-induced interstitial loops; (b3) free ITB migrates due to the interaction force between interstitials in ITB and vacancies inside grain; and (b4) the ITB’s migration leads to the shortening of the twin lamellae’s length.

The structure of fixed ITBs and free ITBs is different and their difference results in various migration behaviors under radiation. The schematic figures for describing the interaction between radiation-induced interstitial loops and these two types of ITBs are plotted in Figure 5. Previous studies have reported that when vacancies appear in the vicinity of grain boundaries (GBs), two mechanisms were proposed to understand the recombination behaviors of these vacancies and interstitials: (I) the captured high-density interstitials could diffuse along the GBs and annihilate the adjacent radiation-induced SFTs [7,31,32]; (II) the interaction force between interstitials in the GBs and vacancies inside grain could lead to the migration of GBs [30,31].

Figure 5(a1–a4) shows the capture process of fixed ITBs to the interstitial loops. Previous studies reported that ITBs could absorb defect clusters in ion-irradiated metals [13,30]. Figure 5(a1) shows the process of an interstitial loop absorbed by fixed ITBs labeled by red solid lines. Many simulation studies concluded that the interstitial clusters firstly prefer to migrate along the ITBs due to the stress gradients of GBs [31]. For fixed ITBs, high-density interstitials are stored after the absorption of fixed ITBs to interstitials and interstitial loops. The continuous vertical ITBs (fixed ITBs) provide diffusion paths for interstitials and their clusters, thus as Figure 5(a2) shows, the absorbed interstitials could diffuse along the fixed ITBs. Figure 5(a3) shows the process of the captured interstitials in fixed ITBs interacting with vacancies and vacancies loops adjacent to grain boundary in the grain. SFTs are a dominant type of vacancy clusters in various irradiated fcc metals such as Ag, Au, Cu and Ni in the absence of noble gases in the materials (most Kr ions penetrated directly through the specimen during radiation) [7,13,27–29], and SFTs are notoriously stable defect clusters in fcc metals and their removal typically requires high-temperature annealing, injection of interstitials, or interaction with mobile dislocations.

However, recent studies showed that the partial dislocations at the ITBs can unlock the stair-rod dislocations of SFTs, resulting in the capturing of SFTs [7]. Another *in situ* study reported that SFTs which are located at the adjacent area of ITBs could migrate to the ITBs and be gradually absorbed by ITBs [30]. Therefore, as shown in Figure 5(a3), these fixed ITBs could capture the vacancy defects such as SFTs and vacancy defects and could annihilate the captured high-density interstitials and partial dislocations in the fixed ITBs. As a result, as Figure 3(a1–a3) shows, the fixed ITBs keep stable after absorbing radiation-induced interstitial loops.

Figure 5(b1–b4) illustrates free ITBs interacting with ion irradiation-induced defect clusters. The free ITB is labeled by a green line in Figure 5(b1). Figure 5(b2) shows the absorption of the free ITB to a defect cluster. For free ITBs, as the boundary of the twin lamella is surrounded by a matrix, the self-interstitial atoms captured by twin lamella are not preferred to diffuse along free ITBs and thus these interstitials accumulate in the ITBs, as Figure 5(b3) shows. The interaction force between interstitials in ITB and vacancies inside grain is large enough to drive the migration of free ITBs. Figure 5(b3) and (b4) shows that the ITB’s migration leads to a decrease in length of the twin lamella. The migration of free ITBs results from the interaction force between the interstitials in ITBs and vacancies in grain by driving the glide of Shockley partial dislocations in the boundary.

Figure 4(b) shows that the ITBs with twin thickness of 3.3 and 5.8 nm have an incubation period after ion irradiation and begin to migrate at the dose of 0.06 dpa for 3.3 nm and at dose of 0.08 dpa for 5.8 nm. But for the ITB with twin thickness of 11.4 nm, it has incubation and doesn’t migrate until the dose arrives at 0.152 dpa. This indicates that the free ITBs with thinner twin lamellae need less incubation time than those with thicker twin lamellae. The incubation can be understood as follows. Interaction force between interstitials in free ITB and vacancies inside grain drives the migration of free ITBs. As the twin thickness increases, free ITBs will need more time to capture radiation-induced defects to induce enough driving force for migration and thus exhibit longer incubation time.

We conclude in the foregoing paragraphs that free ITBs can migrate under ion irradiation, and their migration distances depend on twin thickness. When the twin thickness is smaller than 3 nm, the migration of free ITBs is mainly through stress-driven glide of mobile Shockley partial dislocations, and the stress arises from radiation processes. As the twin thickness increases (smaller than 8 nm), the free ITBs migrate via both the stress-driven glide of mobile Shockley partial dislocations and dislocation climb by absorbing self-interstitial atoms or vacancies, and migration distance decreases. As the twin thickness is larger than 8 nm, the interaction force between Frank partial in ITBs and radiation-induced defects in grain is not large enough to drive the migration of the free ITBs, thus the free ITBs mostly keep stable during ion irradiation.

The migration of free ITBs is the reason for the detwinning of NT Cu film and is detrimental for the application of NT metals. In contrast, previous studies showed that detwinning could be also induced by the migration of CTB either under mechanical deformation or under ion irradiation [8,19,20]. In this study, we observed that the migration of the CTB leads to the reduction of twin thickness in the twin lamella; however, we found and clarified that the fundamental reason for detwinning is the migration of free ITBs.

**Figure 6.**
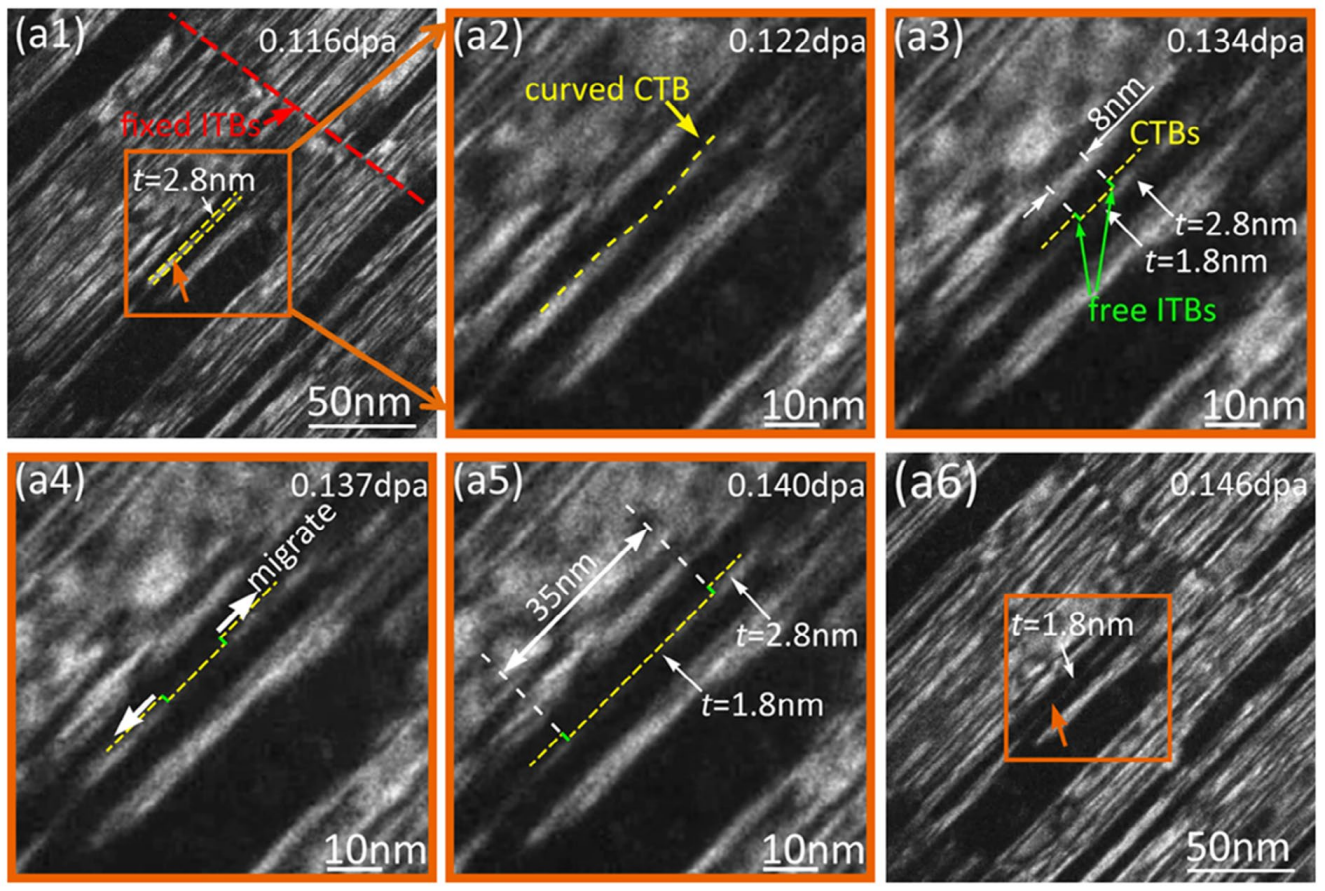
A series of dark-field (DF) TEM snapshots of ion-irradiated Cu film were captured over a dose range of 0.116–0.146 dpa. (a1–a6) show that the interstitial loops were captured by the CTBs of a fixed twin lamella with twin thickness of ~2.8 nm and then the twin thickness decreases to ~1.8 nm. CTBs and fixed ITBs are labeled by yellow and red lines in (a1). (a2–a5) and are the enlarged images of the region labeled by the orange box in (a1), showing the decrease process of twin thickness (*t*). (a6) shows a DF TEM image of the twin lamella at the dose of 0.146 dpa. Compared with Figure 6 (a1), the thickness of part of twin lamella obviously decreases from 2.8 nm to 1.8 nm (about 35 nm wide in the whole twin lamella), leading to the thinning of twin lamella. The figures without any annotations and optional lines are listed as the supplementary material.

Figure 6 shows a series of dark-field (DF) TEM snapshots of *in situ* ion-irradiated Cu film with a dose range of 0.116–0.146 dpa (see Supporting Video V2). Figure 6(a1–a6) shows that a twin lamella with fixed ITB has twin thickness of 2.8 nm at 0.116 dpa in Figure 6(a1) and the thickness of its part decreases to 1.8 nm in Figure 6(a6) over the dose range from 0.116 to 0.146 dpa. Fixed ITBs and CTBs are labeled by red and yellow lines in Figure 6(a1), and the twin thickness is 2.8 nm at the dose of 0.116 dpa. In Figure 6(a2–a5) are the enlarged images of the region labeled by the orange box in Figure 6(a1), and show the decrease process of twin thickness (*t*). Figure 6(a2) shows that the curved CTB was observed with the increase in dose to 0.122 dpa, and Figure 6(a3) shows that two shoulders formed as dose increases to 0.134 dpa. The two shoulders are considered to be composed of two free ITBs (1 nm high) and one CTB (8 nm wide) segment, which are labeled by green and yellow lines, respectively. Figure 6(a3–a5) shows free ITBs’ migration along the direction labeled by the green arrows in Figure 6(a4), leading to the increase in the width of the formed CTB segment from 8 nm to 35 nm, in the dose range from 0.134 to 0.146 dpa. Figure 6(a6) shows a DF TEM image of the twin lamella at the dose of 0.146 dpa. Compared with Figure 6(a1), the thickness of part of twin lamella obviously decreases from 2.8 to 1.8 nm (about 35 nm wide in the whole twin lamellae), leading to the thinning and detwinning of twin lamella.

**Figure 7.**
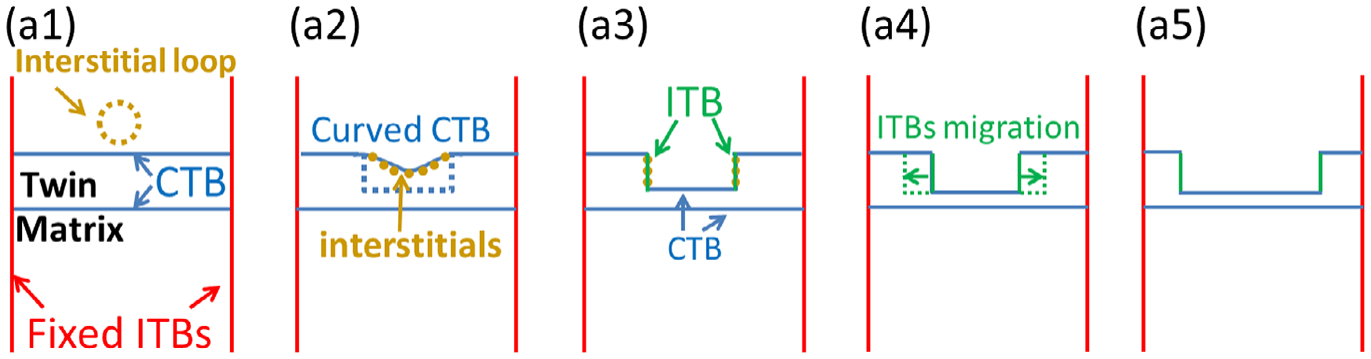
Schematic figures of ion irradiation-induced defects interacting with CTB. (a2) The stress field of the interstitial loop creates curvature in adjacent CTB, and then the CTB loses its identity. (a3) The high-density interstitials in the CTB preferentially diffuse to the two sides and then form two shoulders, which contain two ITBs and one CTB segment. (a4–a5): The ITBs migrate along the directions labeled by green arrows in Figure 6(a4), leading to thinner twin lamella.

Figure 7(a1–a5) shows the schematic figures of a twin lamella thinning and detwinning process. The CTBs are perfectly coherent twin boundaries [7,9,13,30,33–37]. This study has shown that radiation-induced interstitial loops can be easily captured by CTBs [30]. Due to the capture of interstitials by CTB (Figure 6(a1–a2)), high-density interstitials stay at the CTB, and the CTB loses its identity. Figure 7(a1) shows that an interstitial loop was captured by the CTB. Figure 7(a2) shows that the stress field of the interstitial loop creates curvature in the adjacent CTB, and then the CTB loses its identity. Due to the accumulation of interstitials to CTBs, there are relative high-density interstitials at CTBs. Thus, the interaction force of the interstitials at CTBs and vacancies in twin grain drives the glide of Shockley partial dislocations, leading to the migration of the CTB, and the curved CTB now is combined by ITB steps and CTB steps.

Free ITB steps in the curved CTB are unstable and easy to migrate. Studies showed that the vacancy formation energy (*E*_v_) is related to the distance to TBs, and *E*_v_ is significantly lower at TBs than in the perfect crystals of the neighboring [12]. Thus, these free ITB steps prefer to migrate to form two shoulders, as shown in Figure 7(a3). This is similar to the phenomenon that the CTBs could step due to the interaction of glide dislocations with CTBs under *in situ* nanoindentation experiments [38]. The two shoulders are thought to consist of two free ITBs (1 nm height) and one CTB (8 nm width) segment, which are labeled by green and sky-blue lines, respectively. Figure 7(a4–a5) shows that the formed free ITBs migrate along the direction labeled by green arrows in Figure 7(a4) during ion irradiation, leading to a thinner twin layer. Here, the migration behavior of the free ITBs is the same with foregoing discussions of free ITBs’ migration (Figure 5(b1–b4)). Thus, we conclude the foregoing discussion that the migration of free ITBs is the actual mechanism of detwinning of NT metals deduced from CTB’s motion.

## 4. Conclusion

In summary, the mechanism of detwinning in terms of migration behavior of TBs in the nanotwinned Cu film was studied using *in situ* 1 MeV Kr^2+^ ion irradiation at room temperature. The results revealed that the migration of free ITBs is responsible for detwinning while fixed ITBs were stable during radiation. It was suggested that fixed ITBs could provide diffuse paths for point defects, and thus no migration of fixed ITBs was observed during ion irradiation. The migration of free ITBs induced by ion irradiation was observed and resulted in the detwinning of NT metals. Furthermore, although the motion of CTBs leading to detwinning of NT metals was observed, indeed, the migration of free ITBs as a part of CTB was found to be the cause for detwinning. This study could reveal the possible mechanisms of detwinning and may provide further understanding of detwinning mechanisms in nanotwinned metals under ion irradiation.

## Disclosure statement

No potential conflict of interest was reported by the authors.

## Funding

This work was supported by National Magnetic Confinement Fusion Energy Research Project [2015GB121004] from Ministry of Science and Technology of China; National Natural Science Foundation of China [grant numbers 11375018; 11528508; 51231008; 51320105014; 51321003; 51501225; 61377056]; and China University of Petroleum, Beijing [grant number 2462015YQ0602].

## Supplemental data

Supplemental data for this article can be accessed here [https://doi.org/10.1080/14686996.2018.1428877].

## Supplementary Material

Supplementary.zip
