# Supplementary figures and images for: Detwinning through migration of twin boundaries in nanotwinned Cu films under in situ ion irradiation
Source: Sci Technol Adv Mater. 2018 Mar 2;19(1):212–20. doi: 10.1080/14686996.2018.1428877 (PMC5844038; doi:10.1080/14686996.2018.1428877)

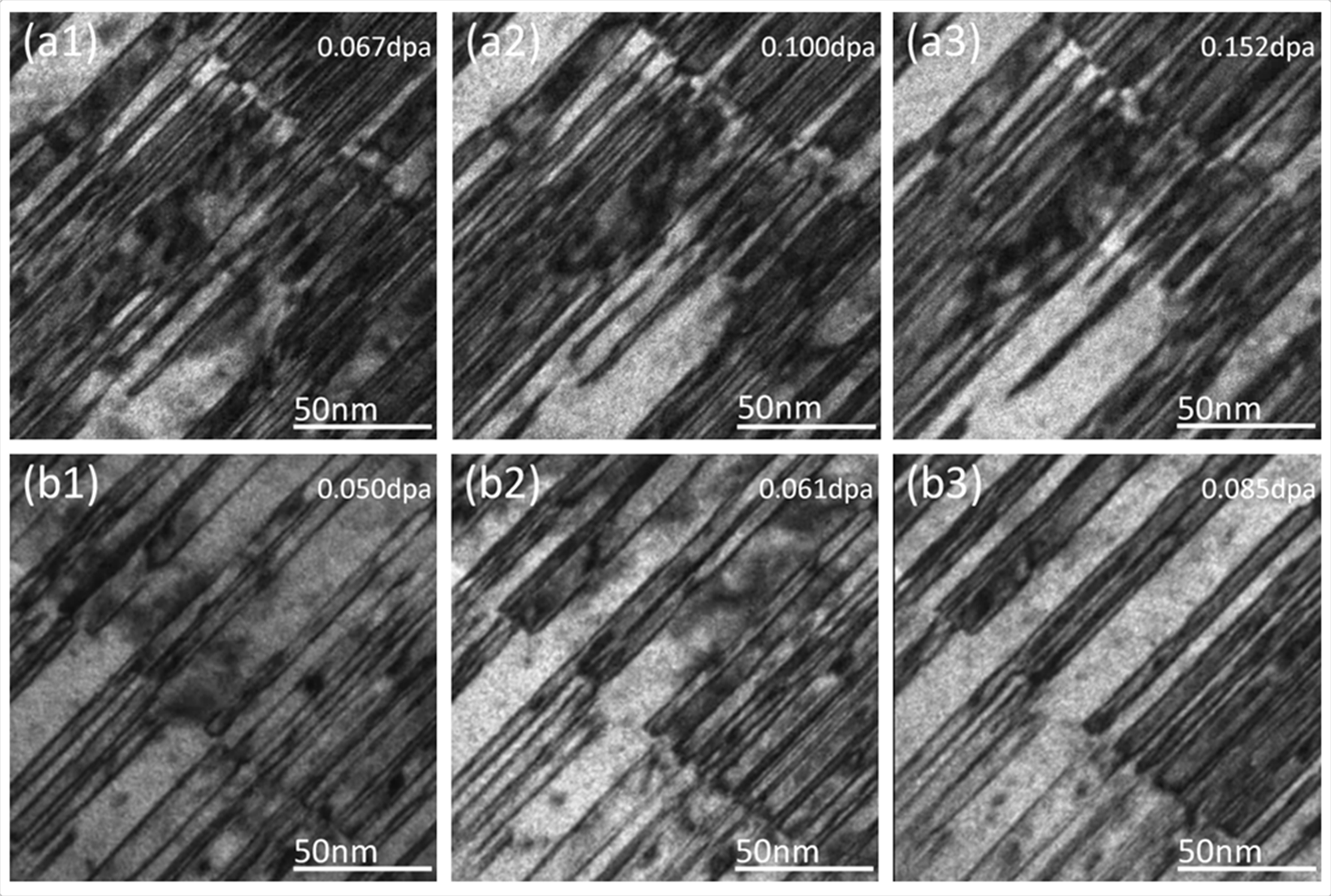

Supplement: Supplementary.zip [file TSTA_A_1428877_SM3500.zip › Figure S1.tif]

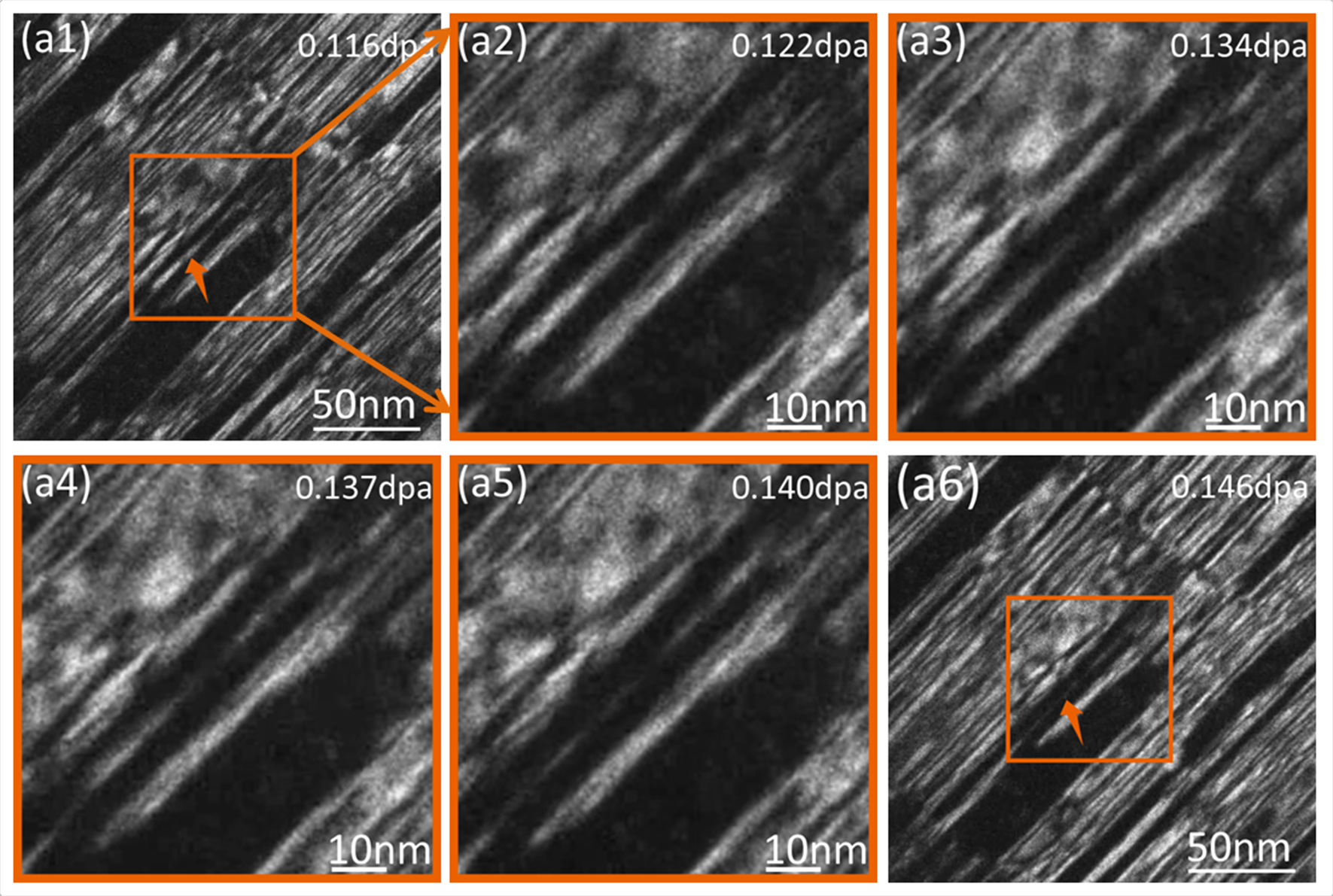

Supplement: Supplementary.zip [file TSTA_A_1428877_SM3500.zip › Figure S2.tif]
